# Mechanisms and consequences of ATMIN repression in hypoxic conditions: roles for p53 and HIF-1

**DOI:** 10.1038/srep21698

**Published:** 2016-02-15

**Authors:** Katarzyna B. Leszczynska, Eva-Leonne Göttgens, Deborah Biasoli, Monica M. Olcina, Jonathan Ient, Selvakumar Anbalagan, Stephan Bernhardt, Amato J. Giaccia, Ester M. Hammond

**Affiliations:** 1 Cancer Research UK and Medical Research Council Oxford Institute for Radiation Oncology, Department of Oncology, The University of Oxford, Oxford, OX3 7DQ, UK; 2 Division of Cancer and Radiation Oncology, Department of Radiation Oncology, Stanford University, Stanford, California 94305, USA

## Abstract

Hypoxia-induced replication stress is one of the most physiologically relevant signals known to activate ATM in tumors. Recently, the ATM interactor (ATMIN) was identified as critical for replication stress-induced activation of ATM in response to aphidicolin and hydroxyurea. This suggests an essential role for ATMIN in ATM regulation during hypoxia, which induces replication stress. However, ATMIN also has a role in base excision repair, a process that has been demonstrated to be repressed and less efficient in hypoxic conditions. Here, we demonstrate that ATMIN is dispensable for ATM activation in hypoxia and in contrast to ATM, does not affect cell survival and radiosensitivity in hypoxia. Instead, we show that in hypoxic conditions ATMIN expression is repressed. Repression of ATMIN in hypoxia is mediated by both p53 and HIF-1α in an oxygen dependent manner. The biological consequence of ATMIN repression in hypoxia is decreased expression of the target gene, *DYNLL1*. An expression signature associated with p53 activity was negatively correlated with DYNLL1 expression in patient samples further supporting the p53 dependent repression of DYNLL1. Together, these data demonstrate multiple mechanisms of ATMIN repression in hypoxia with consequences including impaired BER and down regulation of the ATMIN transcriptional target, *DYNLL1*.

Hypoxia (insufficient oxygen levels) occurs in the majority of solid tumors due to aberrant and inefficient vasculature and is a negative prognostic factor for patients, irrespective of treatment modality^1^,^2^. The poor clinical outcome linked to highly hypoxic tumors is due to increased aggressiveness and metastatic potential, as well as chemo- and radiotherapy-resistance^3^,^4^. Hypoxia also potentiates genomic instability in tumors through down regulation of the DNA repair pathways, including base excision repair (BER), homologous recombination (HR) and mismatch repair (MMR)^5^,^6^,^7^,^8^,^9^. DNA repair has been shown to be repressed over a range of oxygen conditions which occur in human tumors^10^. The mechanisms of repression of repair are numerous (recently reviewed in^11^) and in some cases have been shown to be dependent on the hypoxia inducible factor (HIF-1)^12^. In contrast, hypoxia induced ATM activation is restricted to severely hypoxic conditions (<0.1% O_2_), which also induce replication stress^13^,^14^. Hypoxia-induced ATM activation occurs in response to replication stress and in the context of specific chromatin modifications and importantly, in the absence of detectable DNA damage^15^,^16^,^17^,^18^. ATM activation in hypoxia occurs independently of the MRN complex, however, the requirement for alternative specific ATM interacting proteins has not been investigated^13^. As a result of ATM/ATR signaling in hypoxia the p53 tumor suppressor gene is activated and can lead to hypoxia-induced apoptosis^19^.

Recently, the ATM interacting protein (ATMIN, also known as ASCIZ) was described as having a role in the activation of ATM both in the absence of DNA damage (for example in response to NaCl and chloroquine induced hypotonic stress) and in response to replication stress induced by hydroxyurea (Hu) or aphidicolin (APH)^20^,^21^,^22^,^23^,^24^,^25^. These reports concluded that ATMIN is crucial for ATM activation in the absence of DNA damage, while NBS1 takes over this role when double DNA strand breaks are present e.g. in response to ionizing radiation (IR)^21^,^23^,^24^. It has also been suggested that ATMIN is important for the repair of DNA damage induced by agents such as methyl methanesulfonate (MMS) or H_2_O_2_, and is therefore likely to be involved in the BER pathway^26^. Specifically, ATMIN was demonstrated to be necessary for the formation of Rad51 foci in response to MMS treatment, and for recruitment of 53BP1 to foci after both MMS and APH treatment^22^,^26^,^27^,^28^,^29^. A role for ATMIN in maintaining genomic stability was also demonstrated to be important in the suppression of B cell lymphoma^30^. Finally, ATMIN, which includes a Zn^2+^ finger domain, has been shown to act as a transcription factor driving expression of a very limited number of target genes including *DYNLL1,* which plays an important role in lung morphogenesis^28^,^29^.

Here, we demonstrate that ATMIN was not required for ATM activation in hypoxia and was instead repressed in hypoxic conditions therefore contributing to the hypoxia-mediated repression of DNA repair. The mechanism of repression is complex and is in part dependent on both HIF-1 and p53, demonstrating that repression of ATMIN is critical to the biological response to hypoxia. While this may be due to the role of ATMIN in DNA repair, we also demonstrate for the first time that expression of the ATMIN target gene *DYNLL1* is altered in response to hypoxia.

## Results

### ATMIN is dispensable for ATM activation in hypoxia

**Figure 1:**
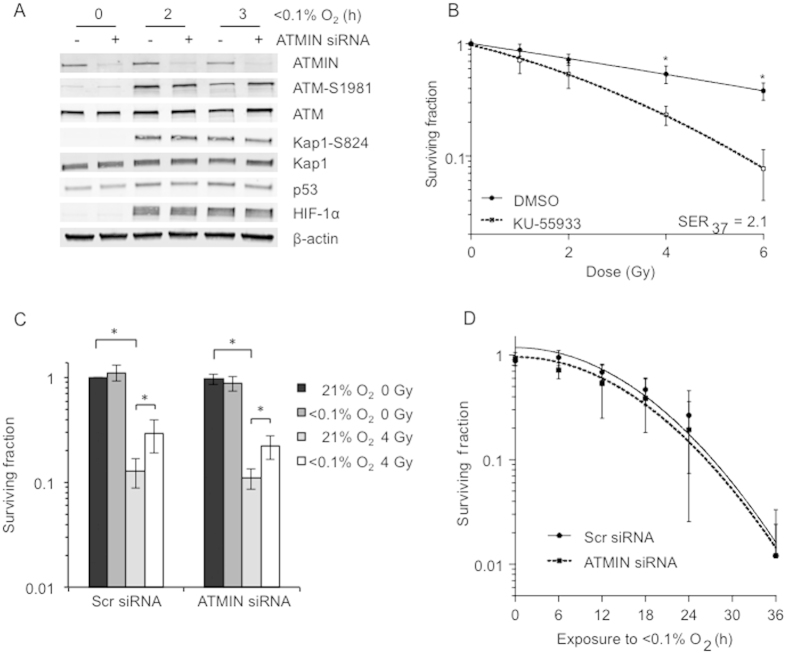
ATMIN loss does not impair ATM activation in hypoxia. (**A**) RKO cells were transfected with either non-targeting scrambled siRNA or ATMIN-specific siRNA. 24 h post transfection, cells were exposed for up to 3 h of hypoxia (<0.1% O_2_). (**B**) Colony survival assay of RKO cells treated with 10 μM KU-55933, exposed to 6 h hypoxia (<0.1% O_2_), and irradiated with 0–6 Gy. Post treatment, cells were allowed to form colonies for 8 days under normal tissue culture conditions. Mean and standard deviation (SD) from 3 independent experiments is shown. (**C**) RKO cells transfected with either scrambled (Scr) siRNA or ATMIN siRNA were exposed to 6 h of hypoxia treatment (<0.1% O_2_) or left at normoxia (21% O_2_), followed by irradiation (4 Gy). Post radiation, cells were reoxygenated and kept under normal tissue culture condition to allow colony formation. (**D**) Colony survival assay of RKO cells transfected with ATMIN siRNA, exposed for up to 36 h of hypoxia (<0.1% O_2_). For all colony formation data mean surviving fractions are shown ± SD from three independent experiments.

To test the hypothesis that ATMIN might be required for hypoxia-induced ATM activation, we used ATMIN siRNA to deplete ATMIN levels in RKO cells. The cells were then exposed to hypoxia (<0.1% O_2_) for 3 h, as we have shown previously that ATM is activated during this period^13^. As expected, a robust induction of phosphorylated ATM (ATM-S1981) and the ATM target Kap-1 (Kap1-S824) was observed in response to hypoxia in the cells treated with the control siRNA. Surprisingly, a similar induction of ATM activity was also observed in the cells with depleted levels of ATMIN (Fig. 1A). To ensure that ATMIN did not have role in sustaining hypoxia-induced ATM activation, experiments over a longer time period were also carried out and again no dependence on ATMIN was observed (Figure S1). This finding was also verified with two single siRNAs to ATMIN (Figure S2). As a control, we verified that loss of ATMIN decreased ATM activity in response to APH-induced replication stress (Figure S3)^22^,^31^. Loss of ATM has a dramatic and well-characterized effect on radiosensitivity, although this has not been as extensively demonstrated under hypoxic conditions^32^. Here, we used an ATM inhibitor (KU-55933) in hypoxic conditions and exposed RKO cells to radiation (0–6 Gy). As expected, the cells treated with the ATM inhibitor were significantly more sensitive to radiation even in hypoxic conditions (SER_37_ = 2.1) (Figs 1B and S4). We then asked if ATMIN might contribute to the role of ATM in radiation response under hypoxic conditions by carrying out the same experiment in cells treated with a siRNA against ATMIN. Loss of ATMIN did not affect the radiation sensitivity of cells irradiated under hypoxic conditions suggesting that ATM function could not have been significantly impacted by ATMIN depletion (Fig. 1C). Previous studies have shown that loss of ATM activity leads to increased sensitivity to hypoxia/reoxygenation^15^,^33^. Therefore, if ATMIN contributes to ATM activity in hypoxia, loss of ATMIN would also be expected to increase sensitivity to hypoxia/reoxygenation. Again, ATMIN was depleted using siRNA in RKO cells and a colony survival assay in response to hypoxia/reoxygenation was carried out. Depletion of ATMIN had no significant effect on cell viability in response to hypoxia compared to wild type RKO cells, which again suggests that ATMIN does not contribute to ATM activity during hypoxia (Fig. 1D). To further support this conclusion, we investigated the induction of apoptosis 6 hours after hypoxic exposure and found that loss of ATMIN did not significantly affect the fraction of apoptotic cells (Figure S5). Altogether, these data demonstrate that ATMIN is not required for the activation of ATM in response to hypoxia-induced replication stress.

### ATMIN is repressed in an oxygen dependent manner through p53 and HIF-1α

**Figure 2:**
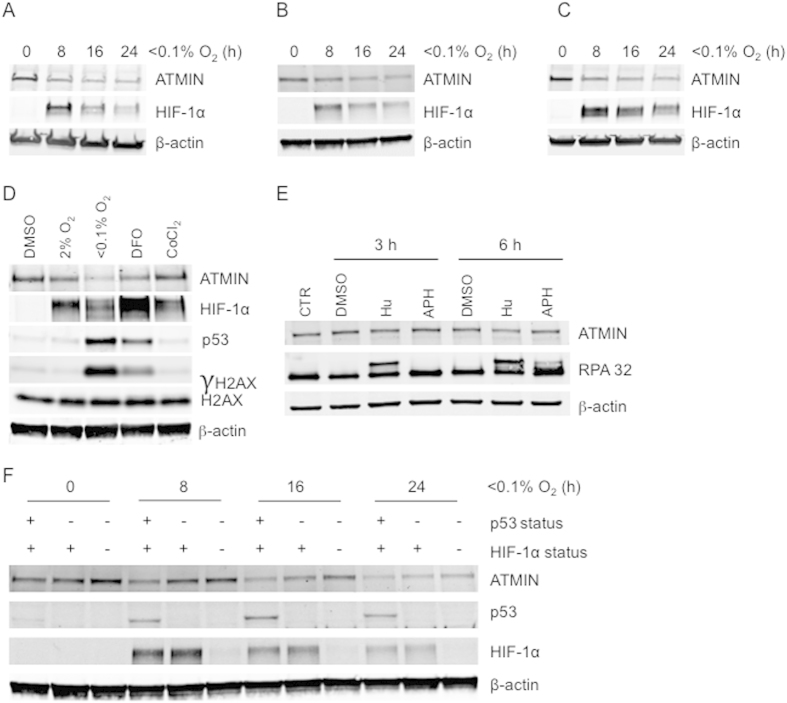
ATMIN expression in hypoxia decreases in an oxygen dependent manner through HIF-1α and p53-dependent pathways. (**A–C**) RKO (**A**), HCT116 (**B**) and RPE1-hTERT (**C**) cells were exposed to hypoxia (<0.1% O_2_) for the times indicated and western blot analysis was carried out using the antibodies indicated (β-actin was used as a loading control). (**D**) RKO cells were treated as indicated for a period of 16 h. The hypoxia mimetic drugs were used as follows; 100 μM DFO and 150 μM CoCl_2_. Western blots using the antibodies indicated are shown. (**E**) RKO cells were exposed to DMSO, 1 mM Hu or 5 μg/ml APH for the indicated amount of time and western blot analysis was carried out for ATMIN, RPA 32 and β-actin (loading control). (**F**) HCT116 p53^−/−^ cells were transfected with either HIF-1α siRNA or Scr siRNA and HCT116 p53^+/+^ cells were transfected with Scr siRNA. Cells were exposed to hypoxia (<0.1% O_2_) for the times indicated and protein lysates analyzed by western blotting with the antibodies shown.

While investigating the potential role of ATMIN on ATM activation in hypoxia we noticed that ATMIN levels decreased over time in hypoxia (Figure S1). Both cancer (RKO, HCT116) and non-cancerous (RPE1-hTERT) cell lines were exposed to hypoxia (<0.1% O_2_) for up to 24 h and analyzed for ATMIN expression by western blotting. In each case the levels of ATMIN protein were significantly decreased in hypoxia (Fig. 2A–C). Next, we tested the oxygen dependency of ATMIN decrease by comparing exposure to severe (<0.1% O_2_) and mild hypoxia (2% O_2_). The levels of ATMIN decreased at both oxygen tensions, although more profoundly at <0.1% O_2_ (27% and 55% decrease at 2% O_2_ and <0.1% O_2_, respectively, when compared to ATMIN levels at 21% O_2_) (Fig. 2D). In addition, we investigated the effect of commonly used hypoxia mimetics, desferoxamine (DFO) and CoCl_2_, and found that DFO treatment partially repressed ATMIN (25% decrease), while CoCl_2_ had no effect on ATMIN protein (Fig. 2D). As the levels of ATMIN have not been reported to change in response to stress previously, we asked if this also occurred upon exposure to alternative sources of replication stress. RKO cells were exposed to Hu or APH for a period of 3–6 h and the levels of ATMIN protein determined. No apparent decrease in ATMIN was detected in response to either treatment suggesting that hypoxia has a unique effect on ATMIN levels (Fig. 2E). To rule out repression of ATMIN with slower kinetics compared to hypoxia, no decrease in ATMIN expression was observed when cells were treated with Hu and APH for up to 18 h (Figure S6). Together, this data demonstrate that ATMIN is repressed in response to a range of hypoxic conditions (<0.1–2% O_2_) but this effect is more profound at the more severe level of hypoxia.

As HIF-1 has been demonstrated to play a role in the repression of other DNA repair proteins in hypoxia we investigated a potential role for HIF-1 in ATMIN repression^11^. However, as the repression of ATMIN was more significant in severe hypoxia compared to milder levels we also included p53 in our analysis, as p53 activity is also restricted to severely hypoxic conditions^34^. We used isogenic cell lines HCT116 p53^+/+^ and p53^−/−^ or RKO HIF-1α^+/+^ and RKO HIF-1α^−/−^ to initially investigate this hypothesis. We observed that ATMIN repression in hypoxia was partially reversed when either p53 or HIF-1α were deleted (Figure S7A,B). To investigate further the contribution of p53 and HIF-1 to ATMIN repression, we used siRNA to knock down HIF-1α in a p53 null background and compared ATMIN protein levels after hypoxia. We observed that the combined depletion of p53 and HIF-1α had the largest effect on rescuing ATMIN protein levels when compared to knock-down or deletion of either transcription factor alone (Fig. 2F). These results suggest that both p53 and HIF-1α independently contribute to repression of ATMIN in hypoxia.

### Hypoxia represses ATMIN expression at the protein level without increasing degradation rates

**Figure 3:**
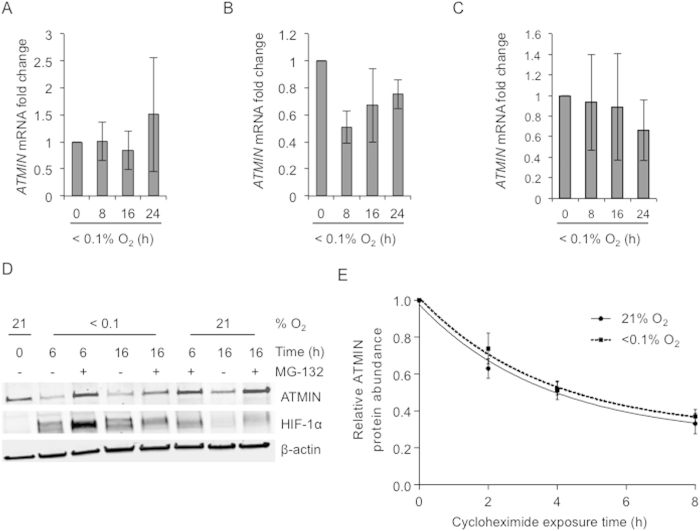
Hypoxia represses ATMIN without increasing degradation (**A–C**) RKO (**A**), HCT116 (**B**) and U-87 (**C**) cells were exposed to hypoxia (<0.1% O_2_) for the times indicated and *ATMIN* mRNA expression was analyzed by qPCR in relation to 18S (reference gene). The mean mRNA expression ± SD from three independent experiments is shown. (**D**) RKO cells were treated with DMSO or 5 μM MG-132 under normoxic (21% O_2_) or hypoxic (<0.1% O_2_) conditions for the times indicated and ATMIN levels were analyzed by western blotting. (**E**) RKO cells were treated with 25 μg/mL cycloheximide under normoxic (21% O_2_) or hypoxic (<0.1% O_2_) conditions for the times indicated. ATMIN protein expression was measured by western blotting and quantified in relation to β-actin levels. The mean ATMIN protein expression ± SD from three independent experiments is shown.

As both transcription factors HIF-1 and p53 play a role in ATMIN repression we investigated *ATMIN* mRNA expression in hypoxia. *ATMIN* mRNA was measured by qPCR in response to hypoxia in 5 cells lines (RKO^p53wt^, HCT116^p53wt^, U87^p53wt^, MCF-7^p53wt^ and U2OS^p53wt^) all of which have a normal HIF-1 response. ATMIN mRNA levels were not significantly affected by hypoxia in the majority of the cell lines tested with the exception of HCT116 cells where mRNA decreased initially (after 8 h) and then basal levels were restored. These results demonstrate that in the cell lines tested, neither p53 nor HIF-1 directly or indirectly repress ATMIN transcription (Figs 3A–C and S8A,B). This was further confirmed by analyzing ATMIN mRNA expression in cells lacking either p53 (HCT116 p53^−/−^) or HIF-1α (RKO HIF-1α^−/−^), again no hypoxia-dependent increase in expression was observed (Figure S8C,D).

ATMIN contains a C-terminal PEST domain, which is associated with proteasomal degradation^21^. Given the stability of ATMIN mRNA in hypoxia we hypothesized that ATMIN could be repressed through increased protein degradation in hypoxia. We measured ATMIN in cells exposed to a proteasomal inhibitor (MG-132) either in normoxia or in hypoxia. The hypoxia-mediated repression of ATMIN was abrogated by the addition of proteasomal inhibitor MG-132 confirming that ATMIN levels are regulated by proteasomal degradation (Fig. 3D). However, ATMIN also accumulated in normoxic conditions (21% O_2_) in the presence of MG-132, suggesting that ATMIN is not specifically targeted to the proteasome in hypoxic conditions. To verify this further we measured the half-life of ATMIN in both normoxia and hypoxia by blocking protein translation with cycloheximide. We determined that the half-life of ATMIN (approximately 4 hours) was not altered by exposure to hypoxia (Figs 3E and S9). Together, these findings suggest that in response to hypoxia ATMIN translation is repressed as opposed to altered transcription or degradation rates.

### Biological consequences of hypoxia-mediated repression of ATMIN include the down regulation of ATMIN target *DYNLL1*

**Figure 4:**
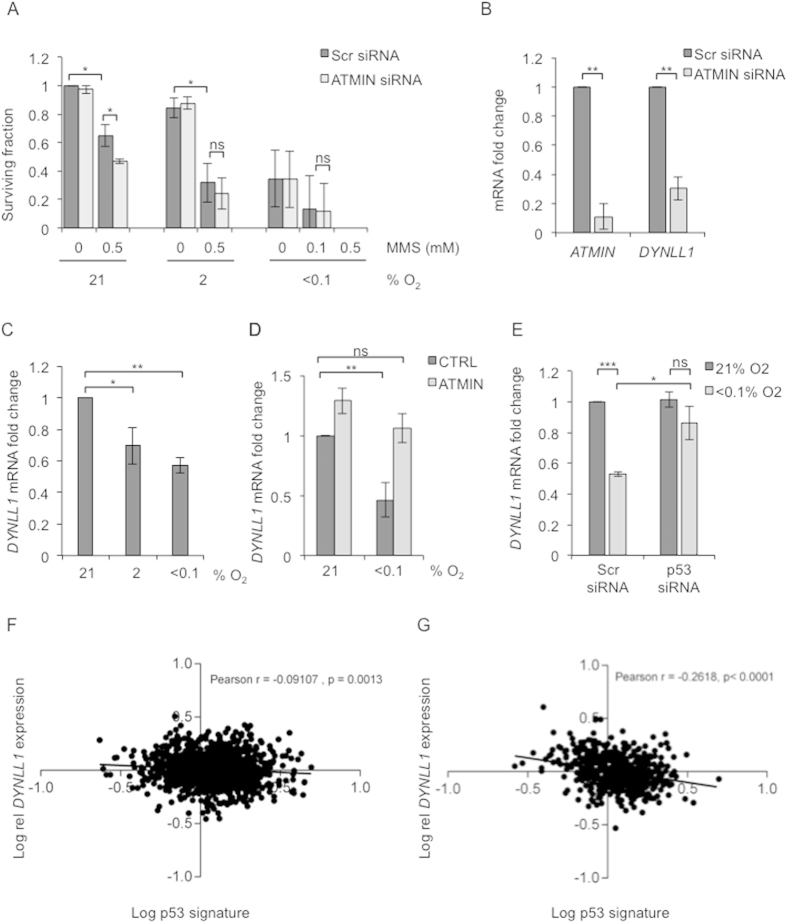
Loss of ATMIN contributes to the hypoxia-mediated repression of DNA repair and a decrease in *DYNLL1* expression. (**A**) RKO cells transfected with either Scr siRNA or ATMIN siRNA were exposed to 20 h of varying oxygen concentrations (21%, 2% and <0.1% O_2_) followed by 1 h of MMS treatment (0.1–0.5 mM as indicated) at the same oxygen tension. Cells were then reoxygenated and the media replaced. Colonies were allowed to form at 21% O_2_ over a period of 8–10 days. Mean surviving fractions are shown ± SD from three independent experiments. (**B**) RKO cells were transfected with either Scr or ATMIN siRNA and expression of *ATMIN* and *DYNLL1* mRNA was analyzed by qPCR at 48 h post transfection. (**C**) *DYNLL1* mRNA expression was analyzed by qPCR in RKO cells exposed to 16 h of varying oxygen concentrations (21%, 2% and <0.1% O_2_). (**D**) RKO cells were transfected with pMSCV-ATMIN vector or an empty vector control. 24 h later cells were transferred to hypoxic conditions (<0.1% O2) for 8 h. qPCR was then carried out for *DYNLL1*. (**E**) *DYNLL1* mRNA was analyzed by qPCR in RKO cells transfected with either Scr or p53 siRNA and exposed to 16 h of hypoxia (<0.1% O_2_). All qPCR bar graphs show mean mRNA expression ± SD from three independent experiments. (**F,G**) Expression of *DYNLL1* (Log_10_ conversion) in the breast (**E**) and lung (**F**) TCGA datasets is shown against hypoxia-inducible p53 signature (Log_10_ conversion). One-tailed p value is shown on each graph for each Pearson r (correlation coefficient).

ATMIN has been described as having a role in BER although this role is somewhat unclear^26^,^27^. BER is known to be functionally repressed in hypoxic conditions and consequently hypoxic cells are less able to repair damage induced by agents such as MMS^5^. To determine if the loss of ATMIN expression contributes to the increased sensitivity to MMS observed in hypoxic cells we exposed cells treated with ATMIN siRNA to range of hypoxic conditions and MMS and carried out a colony survival assay. In agreement with previous reports we observed an increased sensitivity of hypoxic cells to MMS^5^. We also found a significant decrease in cell survival of cells treated with MMS when ATMIN levels were depleted (Fig. 4A)^22^,^26^,^27^. This effect was decreased in cells exposed to 2% O_2_ and completely abolished in cells treated with <0.1% O_2_ (Fig. 4A), where ATMIN was most repressed as a result of the hypoxia exposure.

ATMIN directly transactivates *DYNLL1*, therefore suggesting the novel hypothesis that *DYNLL1* mRNA levels might be regulated in an oxygen dependent manner^29^. In agreement with previous reports, we confirmed that *DYNLL1* expression is dependent on ATMIN levels in RKO cells (Fig. 4B). Next, we exposed cells to hypoxia, and observed an oxygen-dependent down-regulation of *DYNLL1* expression, again with a more profound decrease at <0.1% O_2_ (Fig. 4C), which correlates with ATMIN levels (Fig. 2D). The hypoxia-mediated decrease in *DYNLL1* expression was rescued by over-expression of ATMIN in these conditions (Figs 4D and S10). As the decrease of ATMIN in severe hypoxia was p53 dependent (Figs 2F and S7A), we asked if *DYNLL1* was also regulated in a p53-dependent manner in hypoxia. Therefore we tested the levels of *DYNLL1* mRNA in RKO cells treated with p53 siRNA. As predicted, hypoxia-mediated repression of *DYNLL1* was partially but significantly rescued in the absence of p53 (Fig. 4E). This study highlights an entirely novel link between ATMIN regulation of DYNLL1 and tumor hypoxia.

To date no validated hypoxia signatures, which are specific to the levels of hypoxia which induce replication stress i.e. <0.1% O_2_ exist. However, we recently identified a group of six genes, which are induced by p53 in response to hypoxia (<0.1% O_2_) and can predict p53 status *in vivo* in multiple cancer types^19^. Therefore, we used this group of genes to investigate a potential correlation between *DYNLL1* expression and hypoxic p53 signaling in patient samples. Through analysis of the TCGA breast and lung cancer patient cohorts we found that *DYNLL1* expression significantly and inversely correlated with the hypoxia-inducible p53-dependent group of genes (Pearson r = −0.09197, p = 0.0013 for breast and Pearson r = −0.2618, p < 0001 for lung cancers, respectively) (Fig. 4E), suggesting that hypoxia- and p53-dependent repression of the ATMIN target *DYNLL1* occurs in human cancers. Altogether, our data shows that repression of ATMIN in hypoxia is likely to have multiple consequences, including contributing to the repression of DNA repair pathways and down regulation of the ATMIN target, *DYNLL1*.

## Discussion

**Figure 5:**
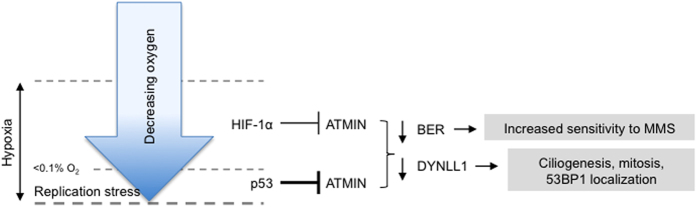
ATMIN expression is repressed in response to hypoxia through mechanisms which include both HIF-1a and p53. The involvement of both HIF-1a and p53 in the repression of ATMIN suggests that this would occur in a significant fraction of the tumour, although p53-mediated repression was more robust. The consequences of loss of ATMIN include increased sensitivity to DNA damaging agents requiring effective BER for repair and decreased expression of DYNLL1. Some of the potential consequences of loss of DYNNL1 in hypoxia are indicated and warrant further study.

Despite having a role in ATM activation in response to replication stress, ATMIN does not play a role in hypoxia-induced ATM activation. Instead, we show for the first time that ATMIN is repressed in response to hypoxia and most significantly under severe hypoxia (<0.1% O_2_). Our data demonstrates that ATMIN repression is in part dependent on both p53 and HIF-1α. The involvement of both transcription factors suggests that ATMIN would be repressed in a broad range of hypoxic conditions and therefore in large areas of tumor. Repression of ATMIN in hypoxia is suggested to contribute to down regulation of BER and decreased expression of *DYNLL1*, as summarized in the proposed model (Fig. 5). Importantly, we show that repression of *DYNLL1* can be inversely correlated with p53 signaling in human cancers suggesting that the consequences of ATMIN repression are likely to play a role in tumors *in vivo*.

The mechanism of repression of ATMIN protein in hypoxia remains unclear, although dependence on p53 and HIF-1α in this process may suggest involvement of micro-RNAs (miRs). Both p53 and HIF-1α are known to induce expression of multiple miRs, which are likely to inhibit protein translation^35^,^36^. Analysis of the *ATMIN* mRNA sequence using available online resources (e.g. www.microrna.org) showed that multiple p53 or HIF-dependent miRs are predicted to bind to the ATMIN transcript, including miR-373 (a HIF-1α target) and miR-34 (a p53 target)^12^,^37^,^38^. In addition, a recent study, which screened for the miR-124 targets involved in the DNA damage response, found ATMIN to be regulated by miR-124^39^. Importantly, miR-124 is a known direct target of p53 suggesting that it is likely to be involved in down-regulation of ATMIN under hypoxia^40^. It is noteworthy that the dependence on p53 and HIF-1 for repression of ATMIN in hypoxia was less significant after longer exposure times to hypoxia (>16 h). This finding suggests that in chronic hypoxic conditions ATMIN might be repressed through an alternative mechanism. A recent study characterized the repression of the BER pathway in chronic hypoxia (72 h 0.2% O_2_) as a consequence of inhibited protein translation^5^. Together, these findings suggest that the repression of ATMIN occurs in hypoxia through mechanisms dependent on HIF-1, p53 and eventual decrease in translation.

Finally, we show that ATMIN repression in hypoxia is linked with down regulation of *DYNLL1* expression. Numerous functions have been attributed to DYNLL1, including regulation of mitosis, spindle orientation, macropinocytosis, nuclear localization/translocation of some proteins e.g. 53BP1, PAK1 or ciliogenesis^41^,^42^,^43^,^44^. Our findings suggest that some or all of these processes will be altered in response to hypoxia through the decreased expression of DYNLL1. In support of this conclusion we have shown that 53BP1 does not form foci in response to hypoxia (<0.1% O_2_) and so there would be no requirement for this function of DYNNL1^13^. Most importantly our data demonstrate that in the absence of functional p53 the hypoxia-mediated repression DYNNL1 described here is alleviated. The specific roles of DYNNL1 in hypoxia driven tumor progression therefore warrant further study.

## Methods

### Cell lines, transfections and drug treatments

RKO, HCT116 (colorectal carcinoma cells), RPE1-hTERT (retinal epithelial cells), HCT116 p53^+/+^ and p53^−/−^ cell lines (Prof Vogelstein, Johns Hopkins^45^), RKO HIF-1α^+/+^ and HIF-1α^−/−^ 46 were cultured in DMEM with 10% FBS. For siRNA knockdown, cells were transfected using Dharma-FECT 1 reagent (Thermo Fisher Scientific) at 50 nM siRNA and used in assays from 24 to 72 h after transfection. Human ATMIN ON-Target SMARTplus siRNA targeting sequences used as a pool or separately: si#1 5′-UUUGAAACAGGACCGAAA-3′; si#2 5′-UUACAACACCACCGAGAUA-3′; si#3 5′-GAUAGAAAGUCCAACGGAU-3′; si#4 5′-CCGAACUGGGCACGAGAUA-3′; were from GE Healthcare Dharmacon (L-020304-01-0005). pMSCV-ATMIN vector was a kind gift from Prof Axel Behrens (The Francis Crick Institute, London) and was transfected using Lipofectamine Ltx reagent (Life Technologies)^24^. Drugs were purchased from Sigma unless otherwise stated and were used at the following concentrations: 100 μM DFO, 150 μM CoCl_2_, 1 mM Hu, 1 μM APH, 25 μg/ml cycloheximide, 5 μM MG-132 or 10 μM KU-55933 (Tocris).

### Colony survival

RKO cells were transfected with 50 nM ATMIN siRNA and the next day re-plated at a density of 250–2000 cells/well in a 6-well plate. After 2 h, cells were exposed to <0.1% O_2_ for 0–36 h and/or drug treatment and then left for 7–9 days to form colonies, which were visualized by crystal violet staining.

### Hypoxia and radiation

Hypoxic treatments at <0.1% O_2_ were carried out in a Bactron chamber (Shel Lab) and at 2% O_2_ in a Don Whitley H35 Hypoxystation. Radiation doses were delivered in a GSM D1 137-Cesium gamma irradiator with at 0–6 Gy doses as previously described^47^.

### Western blotting

Protein lysates were extracted with SDS lysis buffer (10 mM Tris-Cl, pH 7.5, 0.1 mM EDTA, 0.1 mM EGTA, 0.5% SDS, 0.1 mM β-mercaptoethanol, protease/phosphatase inhibitors) and western blotting carried out as described previously^34^. Western blots were imaged using the Odyssey infrared system (LI-COR). The following antibodies were used: ATMIN (Millipore), HIF-1α (BD Biosciences), p53 (Santa Cruz), β-actin (Santa Cruz), ATM (Sigma and Cell Signaling), pATM-S1981 (Epitomics), Kap1-S824 (Bethyl), Kap1 (Bethyl), γH2AX (Millipore) and H2AX (Calbiochem).

### RT-qPCR

Cells were lysed in TRI reagent (Sigma) and mRNA extracted according to manufacturer instructions. The RNA was quantified using NanoDrop. cDNA was synthesized using 500 ng RNA and the Verso cDNA Enzyme kit (Life Technologies). qPCR was carried out with SYBR mix using a 7500 Fast Real-time PCR Detection System (Applied Biosystems). Primer sequences: ATMIN forward: 5′-AACAGCACTGCAGTCTCACA-3′; ATMIN reverse: 5′-CTGGTCTAGGGATTGGTTGGT; 18S forward: 5′-GCCCGAAGCGTTTACTTTGA-3′; 18S reverse: 5′-TCCATTATTCCTAGCTGCGGTATC-3′; DYNLL1 forward: 5′-AGATGCAACAGGACTCGGTG-3′; DYNLL1 reverse: 5′-CCACTTGGCCCAGGTAGAAG-3′.

### Expression analysis in cancer datasets

Raw RNA-sequencing (RNA-seq) data for 1105 Breast invasive carcinoma and 522 Lung adenocarcinoma tumors were downloaded from the TCGA project (accessed through cBioportal: http://www.cbioportal.org/) on 10^th^ September 2015 and 12^th^ September 2015 respectively. To examine tumor-associated p53-activity (referred to as p53 signature in the figure), raw data for each sequenced gene were rescaled to set the median equal to 1, and p53-activity was quantified by averaging the normalized expression of 6 p53 target genes, associated with hypoxia-induced p53 activity (encoding *BTG2, CYFIP2, INPP5D, KANK3, PHLDA3* and *SULF2*)^19^. Log_10_ conversion of the p53 signature was plotted against Log_10_ conversion of raw data for *DYNLL1* (also rescaled to set the median equal to 1). One-tailed p value shown on each graph for each Pearson r (correlation coefficient).

### Statistical analysis

Statistical analysis of clonogenic assays, mRNA expression and expression analysis in cancer datasets was carried out using Graphpad Prism v6.03 software. For colony survival assays the plating efficiency per condition was determined by dividing the total number of counted colonies by the total number of plated cells. Surviving fractions (SF) were calculated by dividing the plating efficiency of treated cells by the plating efficiency of the control cells. Comparison of groups was done using an unpaired t-test with Welch’s correction. Data was fitted according to the linear quadratic model using the formula SF = e^ −(αD + βD^2^). Sensitizer enhancement ratio (SER) was calculated as SER_37_ = D_0_(without sensitizer)/D_0_(with sensitizer) for the same biological effect at SF = 37%. qPCR data was analyzed on 7500 Software V.2.0.5 (Applied Biosystems) using the ΔΔCt method. An unpaired t-test with Welch’s correction was used. Statistical significance is marked as follows: *p < 0.05, **p < 0.01, ***p < 0.001.

## Additional Information

**How to cite this article**: Leszczynska, K. B. *et al*. Mechanisms and consequences of ATMIN repression in hypoxic conditions: roles for p53 and HIF-1. *Sci. Rep.* **6**, 21698; doi: 10.1038/srep21698 (2016).

## Supplementary Material

Supplementary Information
