# Supplementary material for: Mechanisms and consequences of ATMIN repression in hypoxic conditions: roles for p53 and HIF-1
Source: Sci Rep. 2016 Feb 15;6:21698. doi: 10.1038/srep21698 (PMC4753685; doi:10.1038/srep21698)
Supplement: Supplementary Information [file srep21698-s1.pdf]

**Supplementary Information**

# Mechanisms and consequences of ATMIN repression in hypoxic conditions: roles for p53 and HIF-1

Katarzyna B. Leszczynska[1], Eva-Leonne Göttgens[1], Deborah Biasoli[1], Monica M. Olcina[2], Jonathan Ient[1], Selvakumar Anbalagan[1], Stephan Bernhardt[1], Amato J. Giaccia[2] and Ester M. Hammond[1$]

[1] Cancer Research UK and Medical Research Council Oxford Institute for Radiation Oncology, Department of Oncology, The University of Oxford, Oxford, OX3 7DQ, UK [2]Division of Cancer and Radiation Oncology, Department of Radiation Oncology, Stanford University, Stanford, California 94305, USA

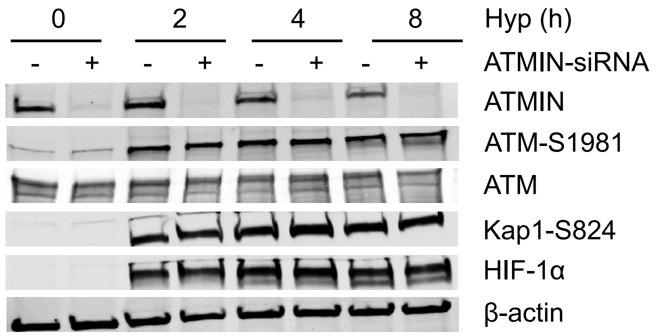


**Supplementary Fig. 1 ATMIN loss does not impair ATM activation in hypoxia**
RKO cells were transfected with either Scr siRNA or ATMIN-specific siRNA. 24 h post transfection, cells were exposed for up to 8 h of hypoxia (<0.1% $O_2$) followed by western blotting for the antibodies indicated.

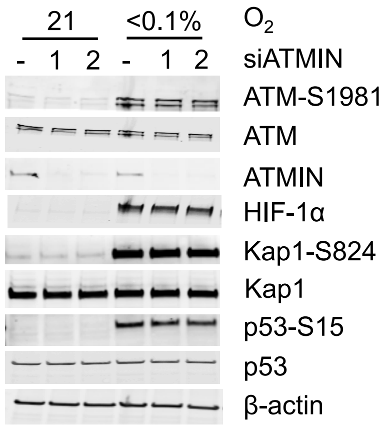


**Supplementary Fig. 2 ATMIN loss does not impair ATM activation in hypoxia**
RKO cells were transfected with either Scr siRNA (-) or 2 different ATMIN-specific siRNAs (1 and 2). 24 h post transfection, cells were exposed to 3 h of hypoxia (<0.1% $O_2$) followed by western blotting for the antibodies indicated.

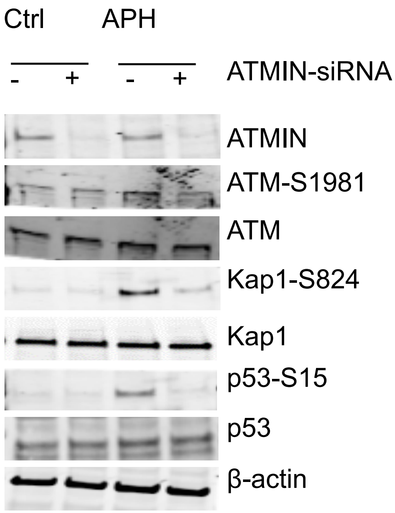


**Supplementary Fig. 3 ATMIN loss impairs Aphidicolin-dependent ATM activation in RKO cells.**

RKO cells were transfected with either Scr siRNA or ATMIN-specific siRNA. 24 h post transfection, cells were exposed to 16 h of 1 μM aphidicolin (APH), followed by western blotting for the antibodies indicated.

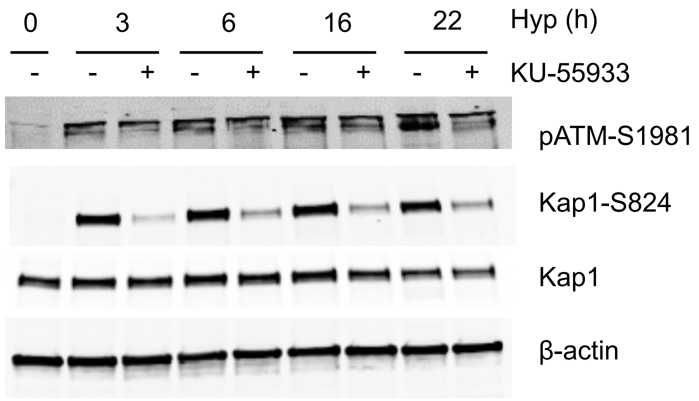


**Supplementary Fig. 4 ATM inhibition with KU-55933 in hypoxia**
RKO cells were treated with KU-55933 in hypoxic conditions (<0.1% $O_2$) for the times indicated followed by western blotting with the antibodies shown.

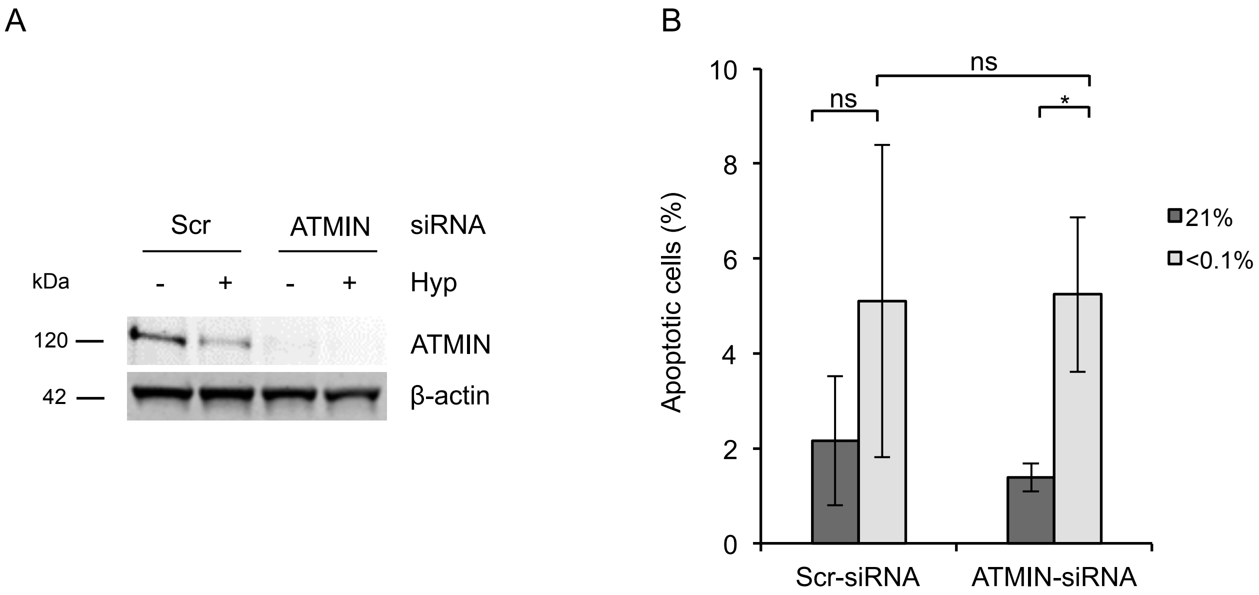


**Supplementary Fig. 5 ATMIN loss does not alter apoptosis in hypoxia**

A. RKO cells were transfected with either Scr or ATMIN siRNA and 48 h later exposed to hypoxia (<0.1% $O_2$) for 8 h. Protein lysates were analyzed by western blotting with the antibodies indicated.

B. Cells from A were fixed and stained with DAPI. Apoptosis was assessed based on nuclear morphology. The bar graph shows mean ± SD apoptosis combined from 3 independent experiments.

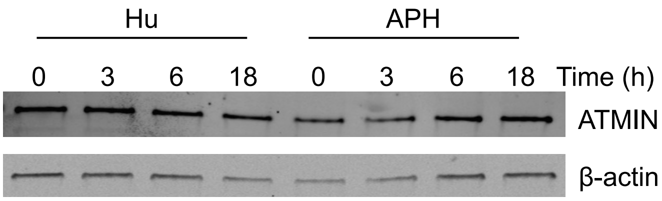


**Supplementary Fig. 6 ATMIN levels do not increase in response to Hu or APH.** RKO cells were exposed to DMSO, 1 mM Hu or 5 μg/ml APH for the indicated amount of time and western blot analysis was carried out for ATMIN and β-actin (loading control).

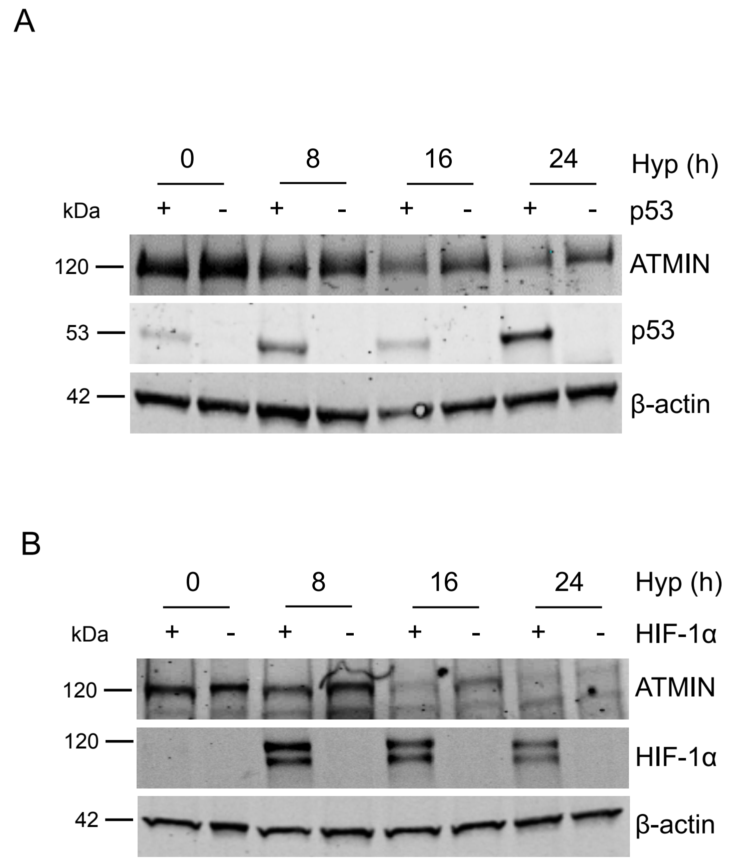


**Supplementary Fig. 7 ATMIN repression in hypoxia is regulated by p53 and HIF-1α**

A. HCT116 $p53^{+/+}$ and $p53^{-/-}$ cells were exposed to hypoxia (<0.1% $O_2$) for the times indicated and ATMIN protein levels were analyzed by western blotting. Blots for p53 and β-actin are shown as controls for p53 status and loading, respectively.

B. RKO HIF-$1\alpha^{+/+}$ and HIF-$1\alpha^{-/-}$ cells were exposed to hypoxia (<0.1% $O_2$) for the times indicated and ATMIN protein levels were analyzed by western blotting. Blots for HIF-1α and β-actin are shown as controls for HIF-1α status and loading, respectively.

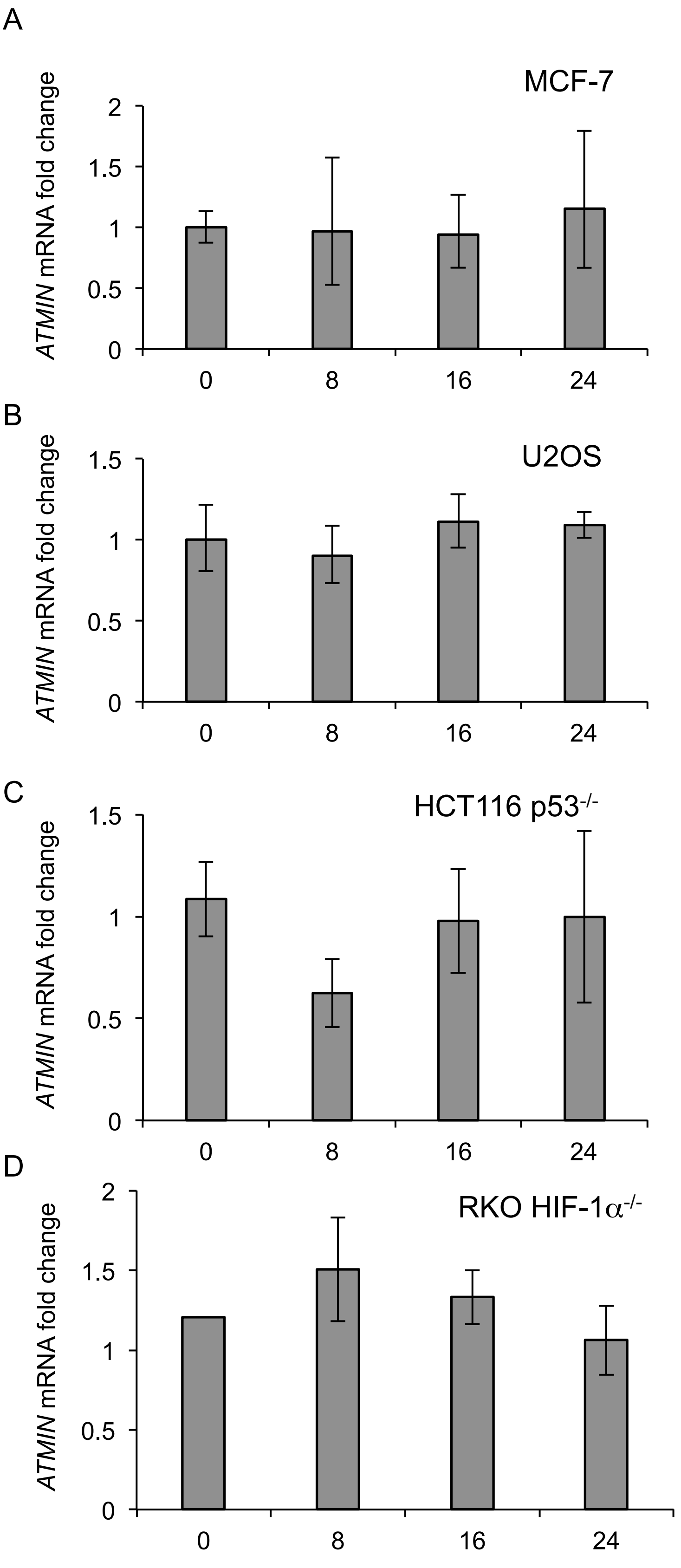


**Supplementary Fig. 8 ATMIN mRNA levels in hypoxia**

A-D. MCF-7 (A), U20S (B), HCT116 p53$^{-/-}$ (C) and RKO HIF-1$\alpha^{-/-}$ (D) cells were exposed to hypoxia (<0.1% $O_2$) for the times indicated and mRNA levels of *ATMIN* were tested by qPCR. 18S was used as a reference gene.

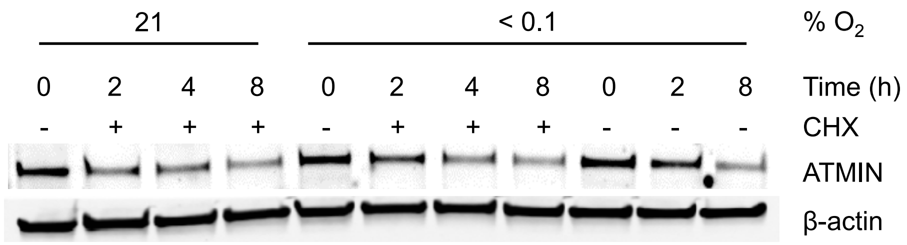


**Supplementary Fig. 9 The half-life of ATMIN is similar under hypoxic and normoxic conditions**

RKO cells were treated with 25 μg/mL cycloheximide under normoxic (21% $O_2$) or hypoxic (<0.1% $O_2$) conditions for the times indicated. ATMIN expression was measured by western blotting and β-actin was used as a loading control.

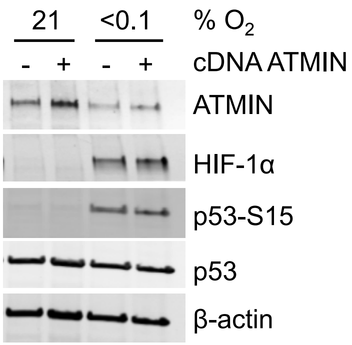


**Supplementary Fig. 10 ATMIN levels goes up with ATMIN cDNA transfection**
RKO cells were transfected with ATMIN cDNA (+) or with empty vector (-) and exposed to hypoxia (<0.1% $O_2$ ) for 8 h followed by western blotting for the antibodies indicated.
